# Ionic Porous Organic Polymers Based on Functionalized Tetraarylborates

**DOI:** 10.3390/polym11061070

**Published:** 2019-06-21

**Authors:** Patryk Tomaszewski, Marcin Wiszniewski, Krzysztof Gontarczyk, Piotr Wieciński, Krzysztof Durka, Sergiusz Luliński

**Affiliations:** Faculty of Chemistry, Warsaw University of Technology, Noakowskiego 3, 00-664 Warsaw, Poland; patrykt94@wp.pl (P.T.); mwiszniewski12@gmail.com (M.W.); krzyksiek@gmail.com (K.G.); pwiecinski@ch.pw.edu.pl (P.W.); kdurka@gmail.com (K.D.)

**Keywords:** ionic organic networks, porous organic polymers, covalent organic frameworks, adsorption, boron

## Abstract

Lithium tetrakis(4-boronatoaryl)borates were subjected to polycondensation reactions with selected polyhydroxyl monomers such as 2,3,6,7,10,11-hexahydroxytriphenylene (HHTP) and 2,3,6,7-tetrahydroxy-9,10-dimethylanthracene (THDMA). Obtained boronate-type ionic porous polymers **TAB1–4** were characterized by PXRD, ^6^Li and ^11^B magic-angle spinning nuclear magnetic resonance (MAS NMR), FT-IR, SEM, and TGA. They exhibit relatively good sorption of H_2_ (up to 75 cm^3^/g STP), whereas N_2_ uptake at 77 K for lower pressure range is relatively poor (up to 50 cm^3^/g STP below *P*/*P*_0_ = 0.8). In addition, the effect of elongation of aryl arms in the tetraarylborate core on the materials’ properties was studied. Thus, it was found that replacement of the 4-boronatophenyl with 4-boronatobiphenylyl group has a negative impact on the sorption characteristics.

## 1. Introduction

Nanoporous ionic organic networks (NIONs) comprise negative or positive charges whereas free counterions are located in pores to maintain electrical neutrality of the structure. The synthesis of nanoporous ionic networks has recently attracted a significant interest due to additional possibilities offered by these materials in comparison to neutral porous polymers. For example, ion exchange or mixing can be used for fine-tuning of the pore size. NIONs may also exhibit improved sorption efficiency and selectivity due to electrostatic interactions of guest molecules with charged pore walls, which may be either attractive or repulsive. Recently, various synthetic strategies towards ionic porous polymer networks have been developed [1]. The direct synthesis from appropriate building blocks seems to be still the most versatile method. However, other approaches such as hard/soft templating method, the ionic complexation method as well as postsynthetic modifications are also in use. The selection and optimization of a synthetic strategy is of course crucial for achieving desired porous structural parameters such as pore shape, pore size, pore distribution profile, etc. The use of precursors possessing a general tetrahedral topology imposes a three-dimensional structure of a resulting polymer network. Many examples of such systems were reported in the recent literature. However, it should be noted that the presented materials featured nonionic structures. Within this group, classical examples include highly porous materials COF-102 and COF-103 based on tetraboronic acids C[*p*-C_6_H_4_B(OH)_2_]_4_ and Si[*p*-C_6_H_4_B(OH)_2_]_4_, respectively [2]. Boron compounds are frequently used as building blocks for the preparation of porous networks with a special emphasis on COFs [3]. However, the majority of them possessed a neutral character whereas examples of boron-based NIONs are scarce [4].

Functionalized tetraarylborates were used by Wuest and coworkers to construct the anionic porous supramolecular networks bearing multiple hydrogen-bonding sites [5]. In 2015, a synthesis of a series of ionic porous polymers via Sonogashira cross-coupling of lithium tetrakis(4-iodophenyl)borate with selected alkynes was reported [6]. The presented materials showed high I_2_ uptakes. Tetrakis(pentafluorophenyl)borate shows higher chemical stability compared to nonfluorinated tetraphenylborate anion and was used as a building block in linear polymers or dendrimers [7,8]. The approach to homogeneous porous polymers via a Yamamoto coupling polymerization of lithium tetrakis(4-bromo-2,3,5,6-tetrafluorophenyl)borate (Li[B(C_6_F_4_Br)_4_]) was unsuccessful. However, it was possible to obtain the conjugated porous copolymer (Li-ABN) by Sonogashira coupling polymerization of Li[B(C_6_F_4_Br)_4_] with 1,3,5-triethynylbenzene [9]. Furthermore, Long and coworkers [10] reported the anionic tetraphenylborate conjugated porous polymers by Sonogashira cross-coupling of tetrakis(4-iodophenyl)borate, tetrakis(4-iodo-2,3,5,6-tetrafluorophenyl)borate, or tetrakis(4-bromo-2,3,5,6-tetrafluorophenyl)borate with 1,4-diethynylbenzene and its tri(ethylene glycol)-substituted derivative. Obtained materials showed good solid-state conductivity and strong ion-conducting transport characteristics. The salt Li[B(C_6_F_5_)_4_]) was subjected to polycondensation with 5,5′,6,6′-tetrahydroxy-3,3,3′,3′-tetramethyl-1,1′-spirobisindane through a dibenzodioxane-forming reaction (C–O bond construction) under mechanochemical conditions resulting in an anionic porous polymer network with Li^+^ cations [11]. It was susceptible to postsynthetic modification involving cation exchange. Another interesting example is a new charge-separated metal–organic framework possessing diamondoid structure assembled from a functionalized tetraarylborate ligand and Cu^+^ cation which showed high CO_2_ vs. N_2_ sorption selectivity [12].

In this contribution, we report on the use of ionic boronated tetraarylborate counterparts of above-mentioned boronated tetraphenylmethane and tetraphenylsilane connectors for the preparation of a series of microporous polymers. Approach to parent precursors comprising tetrakis[4-(dihydroxyboryl)phenyl]borate and tetrakis(4-(dihydroxyboryl)biphenylyl)borate anions has been described in our recent paper [13]. The synthetic part is complemented by comprehensive characterization of newly obtained ionic materials including studies on their porosity aimed at evaluation of their gas sorption selectivity.

## 2. Materials and Methods

### 2.1. General Comments 

THF used for reactions was distilled from sodium/benzophenone ketyl under argon and stored over 4Å molecular sieves. Starting materials including *tert*-butyllithium (1.7 M solution in pentane), trimethyl borate, chlorotrimethylsilane, and 2,3,6,7,10,11-hexahydroxytriphenylene (HHTP) were used as received without further purification. Potassium tetrakis(4-iodophenyl)borate and potassium tetrakis(4′-bromo-4-biphenylyl)borate [13] as well as 2,3,6,7-tetrahydroxy-9,10-dimethylanthracene (THDMA) [14] were prepared as described previously. Syntheses of precursors **1,2** as well as materials **TAB1–4** were performed under argon atmosphere. ^1^H and ^13^C NMR chemical shifts are reported in ppm from TMS with the residual solvent resonances as internal standards. The ^6^Li and ^11^B magic-angle spinning nuclear magnetic resonance (MAS NMR) spectra were recorded with a Bruker II Avance 500 spectrometer (11.74 T; recycle delay of 10 to 30 ms; spinning rate 5 or 10 kHz).

### 2.2. Synthesis

#### 2.2.1. Lithium tetrakis[4-(dimethoxyboryl)phenyl]borate THF solvate (1)

A solution of potassium tetrakis(4-iodophenyl)borate (4.4 g, 5.1 mmol, dried for 1 h at 100 C, 10^−3^ Torr) in dry THF (20 mL) was added to a stirred solution of *t-*BuLi (1.7 M solution in pentane, 26 mL, 44.2 mmol) in THF (60 mL) at −78 °C. A resulting dark-green mixture containing the tetrakis(4 lithiophenyl)borate intermediate was stirred for 1 hr and cooled to −100 °C. Then B(OMe)_3_ (6 mL, 54.5 mmol) was added dropwise at −78 °C. The mixture was stirred for 1 h and allowed to warm to ca. 0 °C. Then Me_3_SiCl (3.5 mL, 28 mmol) was added and the mixture was stirred for 2 h at room temperature. The mixture was concentrated under reduced pressure. To the residue Et_2_O (40 mL) and DCM (40 mL) were added. The solution was removed from over a white precipitate with a syringe to another flask and filtered. Then it was evaporated under reduced pressure to leave a white solid. Yield 3.5 g (76%). ^1^H NMR (400 MHz, DMSO-*d*_6_) δ 7.36 (d, *J* = 7.3 Hz, 8H), 7.15 (m, 8H), 3.68–3.54 (m, 16H), 3.18 (s, 24H), 1.81–1.64 (m, 16H) ppm. ^13^C NMR (101 MHz, DMSO-*d*_6_) δ 135.25, 131.86, 126.61, 67.46, 50.23, 49.03, 25.56 ppm.

#### 2.2.2. Lithium tetrakis[4′-(dimethoxyboryl)-4-biphenylyl]borate THF solvate (2)

This material was prepared using a protocol similar to that described for precursor **1** starting with potassium tetrakis(4′-bromo-4-biphenylyl)borate (4.89 g, 5.0 mmol) [13]. The product was isolated as a pale yellow solid. Yield (4.2 g, 70%). ^1^H NMR (400 MHz, DMSO-*d*_6_) δ 7.98 (s, 4H), 7.83 (d, *J* = 7.9 Hz, 8H), 7.60 (d, *J* = 7.9 Hz, 8H), 7.39 (m, 16H), 4.14 (q, *J* = 5.2 Hz, 8H), 3.63–3.55 (m, 16H), 3.18 (d, *J* = 5.2 Hz, 24H), 1.78–1.70 (m, 16H) ppm. ^13^C NMR (101 MHz, DMSO-*d*_6_) δ 143.77, 136.47, 135.04, 133.99, 129.08, 126.55, 125.47, 124.41, 67.46, 49.03, 25.56 ppm.

#### 2.2.3. Porous materials **TAB1–4**

**TAB1**. In a Schlenk reactor, precursor **1** (0.91 g, 1 mmol) was dissolved in dry THF (10 mL) and then a solution of HHTP (0.44 g, 1.33 mmol) in dry THF (20 mL) was added. The resulting thick slurry was stirred for 3 days at room temperature and filtered under argon. The crude product was washed with several portions of dry THF until the filtrate was almost colorless. The product was dried for 6 hrs at 100°C under reduced pressure (10^−3^ Torr). Yield 0.55 g (59%).

**TAB2**. This material was prepared using a protocol similar to that described for **TAB1** using precursor **1** (0.91 g, 1.00 mmol) and THDMA (0.54 g, 2.00 mmol). Yield 0.60 g (58%).

**TAB3**. This material was prepared using a protocol s1imilar to that described for **TAB1** using precursor **2** (1.21 g, 1.00 mmol) and HHTP (0.44 g, 1.33 mmol). Yield 0.73 g (59%).

**TAB4**. This material was prepared using a protocol similar to that described for **TAB1** using precursor **2** (1.21 g, 1.00 mmol) and THDMA (0.54 g, 2.00 mmol). Yield 0.81 g (60%).

### 2.3. Physicochemical Characterization

#### 2.3.1. Thermogravimetric analysis 

Thermogravimetric analysis (TGA) was performed on a TGA/DSC1 (Mettler-Toledo) system under continuous flow of argon at the ramp rate of 10 K min^−1^ from 30 to 700 °C. The samples of 2–10 mg were prepared in covered ceramic crucibles. An empty crucible was used as a reference. α-Al_2_O_3_ was used for instrument calibration. Samples were measured after conditioning at 150 °C for 1 h. TGA diagrams are placed in the Supplementary Materials (Figures S5–S8).

#### 2.3.2. Scanning electron microscopy studies 

The morphology, size, and shape of COFs materials were determined using scanning electron microscopy (SEM). The samples were observed using SEM/STEM Hitachi s5500 with maximal resolution of 0.4 nm at acceleration voltage of 30 keV and below 2 nm at 1 keV. The observation was performed using secondary electron (SE) signal. The microscope was equipped with Cold Field Emission gun ensuring high resolution at relatively low beam current, which is important in the case of observation of materials sensitive to electron beam, for example, COFs. Additionally, in order to minimize the effect of surface charging as well as to confine the structure damage by electrons, low beam current and low acceleration voltage (3 keV) were applied. Such observation conditions ensured compromise between brightness/resolution and safety for COFs samples. Low accelerating voltage leads also to lower depth of electron penetration which enables more detailed characterization of surface morphology. Moreover, samples were not coated by conductive layer for SEM observation. Such approach ensures that the morphology of the samples was not changed by deposited layer.

#### 2.3.3. Powder X-ray diffraction measurements

Powder X-ray diffraction (PXRD) analyses of **TAB1–4** were carried out on a BrukerAXS WAXS D8 powder diffractometer equipped with Cu radiation source (Cu *K*_α_, λ = 1.54184 Å), a no background sample holder and a VÅNTEC detector. Data were collected over a 2*θ* range of 2.1° to 35° in Bragg−Brentano geometry with a generator setting of 40 kV and 40 mA, step size of 0.02°, and exposure time per step of 10 s. PXRD patterns for all samples are placed in Supplementary Materials (Figures S9–S12).

#### 2.3.4. Sorption measurements 

Micromeritics ASAP 2020 Surface Area and Porosity Analyzer was used to measure the nitrogen adsorption isotherms. Samples were placed in oven-dried tared tubes equipped with filler rod, capped with SealFrit stopper. Samples were activated at maximum temperature of 150 °C in vacuo (0.002 Torr) for 24 h. N_2_ isotherms were measured using liquid nitrogen baths (77 K). Ultrahigh purity grade (99.999% purity) N_2_, H_2_, CO_2_, CH_4_, and He, oil-free valves, and gas regulators were used for the free space correction and measurement. Relative pressure (*P*/*P*_0_) range for BET analysis was selected based on Rouquerol’s criteria [15].

## 3. Results

### 3.1. Synthesis

**Scheme 1:**
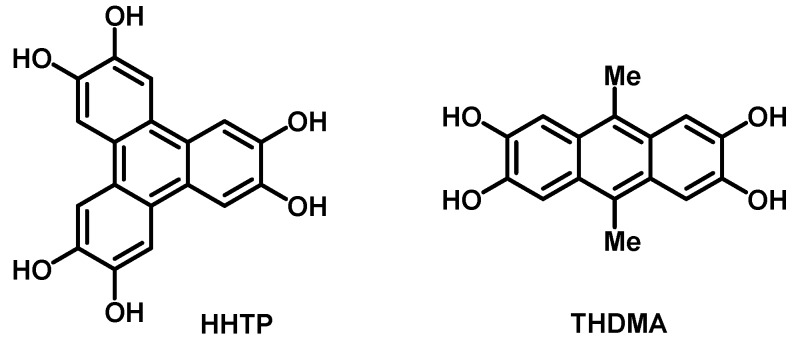
Structures of polyhydroxyl precursors.

In our initial attempts, we used ammonium and potassium tetrakis[4 (dihydroxyboryl)phenyl]borates [13] for polycondensation reactions with polyhydroxyl linkers HHTP and THDMA (Scheme 1). The reactions were performed under standard conditions used for the preparation of boronate-type COFs (mesitylene/1,4-dioxane, 85 °C) [16]. Unfortunately, they suffered from low yields (below 30%) of precipitated polymers, especially in case of using THDMA. In addition, analysis of a material obtained from the reaction with HHTP revealed extensive deboronation of a tetraarylborate scaffold, mostly due to protonolysis of external B–C bonds. Overall, it seems that low conversion of starting materials is due to unfavorable equilibria resulting from the presence of additional amounts of water in the used tetraarylborates. Of course, substantial amounts of water are formed during the polycondensation reactions eventually stopping the progress of the reaction.

**Scheme 2:**
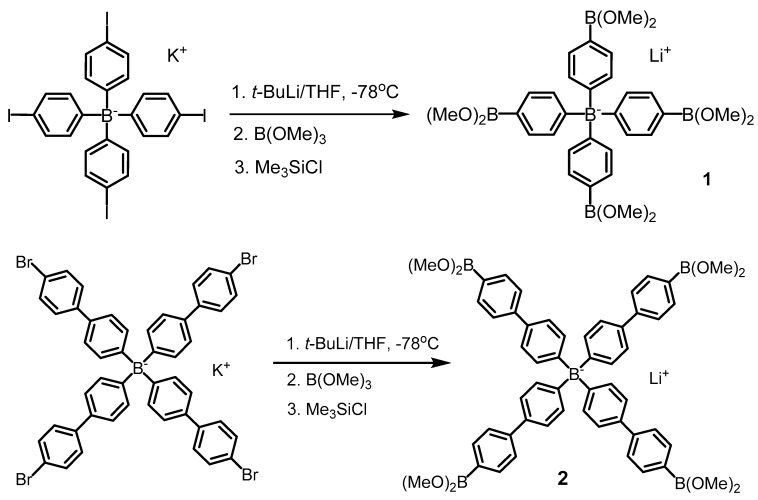
Synthesis of tetraarylborate precursors **1** and **2**.

Therefore, we decided to replace boronic precursors with their boronic ester analogues **1**,**2**. Their synthesis followed the protocol described by us recently. At first, potassium tetrakis(4-iodophenyl)borate was prepared by the reaction of an excess of 4-iodophenyllithium (5 equivs) with BCl_3_ (1 equiv) in Et_2_O [13]. Then, the tetraiodo precursor was subjected to halogen-lithium exchange followed by addition of B(OMe)_3_. However, the final hydrolysis step was omitted. Instead, Me_3_SiCl was added, which resulted in the formation of a solution containing respective lithium tetrakis[4-(dimethylboronato)phenyl]borate **1** (Scheme 2). We have found previously [17] that the use of Me_3_SiCl is a mild and convenient method for the conversion of an ate complex ArB(OMe)_3_Li into the neutral boronate ArB(OMe)_2_. The byproduct LiCl can be separated by filtration whereas Me_3_SiOMe remains in solution and subsequently can be removed under reduced pressure. The analogous protocol was employed for the preparation of compound **2** with longer 4′-biphenylyl arms in the anion structure. The products were obtained as white (**1**) or pale yellow (**2**) solids well soluble in THF.

**Scheme 3:**
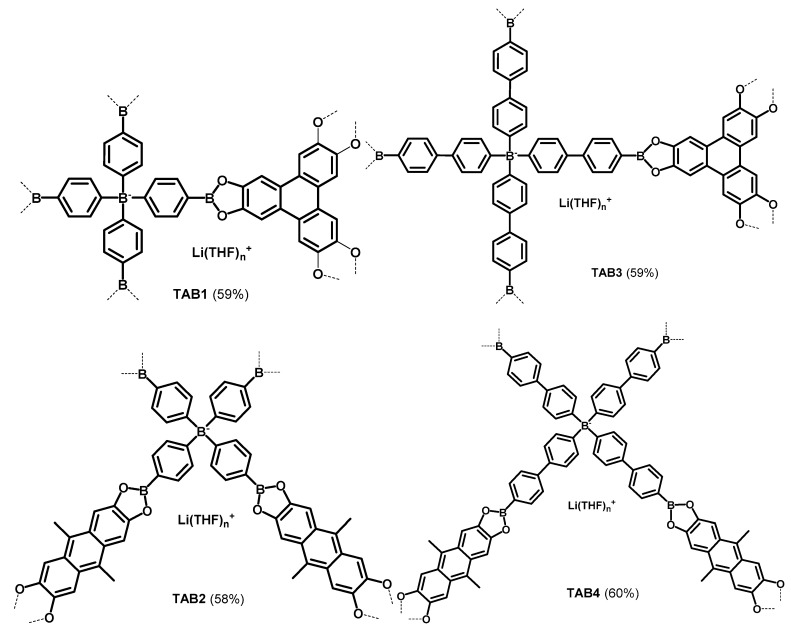
Basic structural motifs of materials **TAB1–4**.

The synthesis of **TAB1–4** involved stirring of a mixture of a precursor (**1** or **2**) with a stoichiometric amount of a polyhydroxyl linker (HHTP or THDMA) using THF as a solvent at 50 °C for 3 days. The gradual precipitation of a voluminous material from a mixture was already observed at the initial period of the reaction (ca. 5–30 min). Obtained products were isolated by filtration under argon atmosphere and washed several times with anhydrous THF until the filtrate was almost colorless. Then, they were dried under reduced pressure at 40 °C to give **TAB1–4** (Scheme 3) as dark colored olive-brown, gray, or violet solids in 58–60% yields. ^11^B NMR spectroscopy analyses of hydrolyzed samples confirmed the presence of two types of boron atoms. In addition, ^1^H NMR spectra of hydrolyzed samples revealed the presence of substantial amounts of THF which is presumably coordinated to lithium cations.

The thermal stability of **TAB1–4** (dried at 150 °C prior to analysis) was investigated under argon atmosphere by TGA technique (Figures S5–S8). All materials are thermally stable in the lower temperature range (30–250 °C) as evident by small sample mass losses. Mass loss of 0.5–2% can be mostly associated with the removal of adsorbed gases. Then continuous mass loss is observed in the range of 250 to 650 °C followed by gentle plateau to 700 °C. In the case of **TAB3,4**, the highest rate of mass loss is observed at ca. 400 °C suggesting that these materials are more prone to thermal degradation compared to **TAB1,2**. In fact, the total mass loss (measured up to 700 °C) is significantly higher for **TAB3,4** (ca. 43%) than for **TAB1** (30%), and **TAB2** (22%).

### 3.2. Morphology and Structural Studies

**Figure 1:**
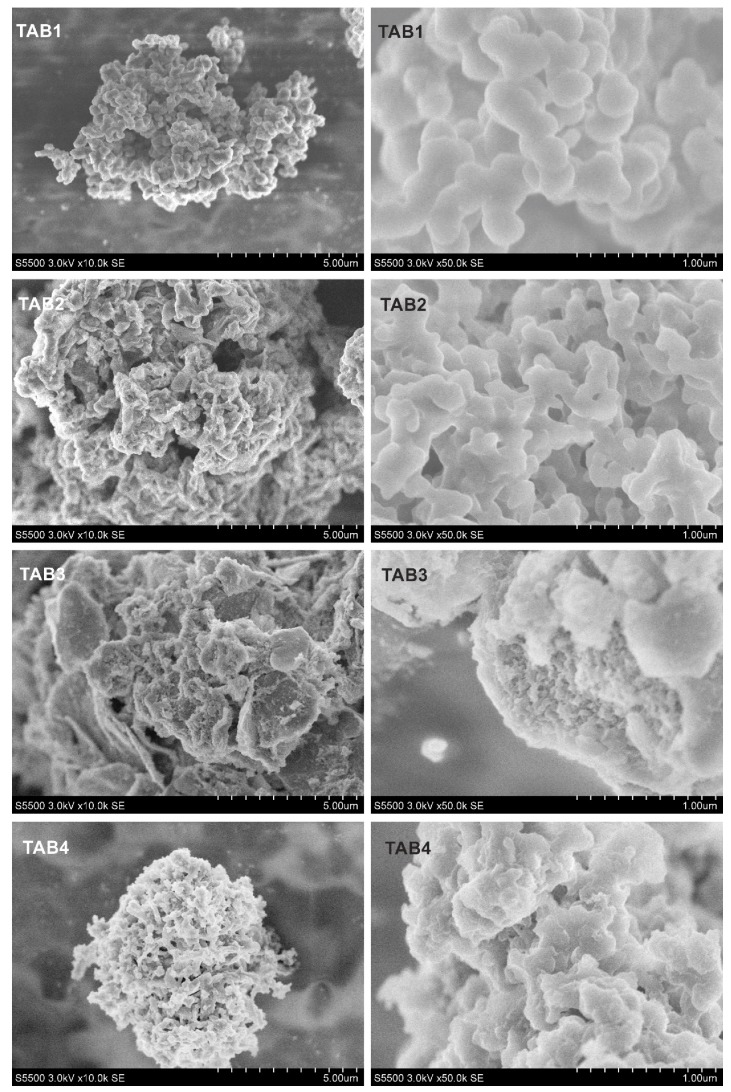
SEM images of TAB1–4.

**Figure 2:**
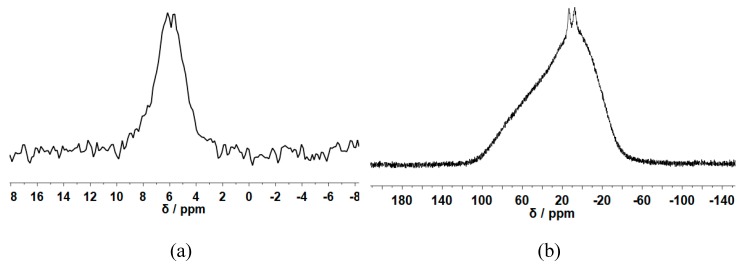
^6^Li (**a**) and ^11^B (**b**) magic-angle spinning nuclear magnetic resonance (MAS NMR) spectra of **TAB1.**

Scanning electron microscopy (SEM) indicates differences in morphology of studied materials (Figure 1). They form agglomerates of irregular shape and different size, from few to even hundreds of micrometers. However, only the smaller agglomerates were observed in detail since they exhibit higher stability during observation. Agglomerates of **TAB1** were composed of a well-formed sphere- and ellipsoid-shaped nanoparticles, approximately 200 nm in diameter. The anthracene-based polymer **TAB2** tends to form larger agglomerates compared to **TAB1**. They are also composed of small nanoparticles which in this case typically adopt rather irregular but generally elongated shapes with length and width dimensions of approximately 500 and 100–200 nm, respectively. In SEM images of **TAB3**, the irregular sponge-like clusters of a few μm size are observed. It seems that they have more compact character relative to agglomerates of **TAB1** and **TAB2**. However, the presence of macropores of ca. 50 nm diameter in their structures is evident. Finally, the morphology of **TAB4** is similar to those observed for **TAB2** and features clusters formed by easily distinguishable nanoparticles of 200–500 nm size. However, their shapes are very irregular compared to those observed for **TAB1** and **TAB2** PXRD analyses (Figures S9–S12) revealed that polymers **TAB1** and **TAB4** are essentially amorphous. The diffractograms of **TAB2** and **TAB3** show a few broadened peaks consistent with some degree of crystallinity but we were unable to provide the reliable structure model which would account for the obtained data. To gain further insight into structure of materials **TAB1–4**, they were investigated using ^6^Li and ^11^B MAS NMR spectroscopy. The spectra for **TAB1** are shown in Figure 2, all data are provided in the Supplementary Materials (Figures S13–S20). Lithium acetate dihydrate was used a reference (δ = 5.80 ppm) for ^6^Li MAS NMR experiments [18]. ^6^Li MAS NMR chemical shifts for **TAB1**, **TAB2**, and **TAB4** were observed in a rather narrow range of 5.9 to 6.2 ppm (half-widths of ca. 200 Hz), which points to tetrahedral coordination of lithium cation with four oxygen atoms similar to that existing in the crystal structure of the reference lithium salt [19]. In case of **TAB3** the major ^6^Li MAS NMR resonance occurs at 8.1 ppm which may indicate different coordination mode of lithium cations compared to **TAB1,2** and **TAB4**. The ^11^B MAS NMR spectra of **TAB1–4** are similar one to another and show very broad resonances (half-widths of ca. 10 kHz), which is strongly indicative of the amorphous character of all studied materials.

FT-IR spectra of **TAB1–4** were recorded using ATR technique (see Supplementary Materials, Figures S21–S24). They feature medium intense or strong bands of B−O stretching vibrations in the range of 1353–1355 cm^−1^. The spectra of **TAB1** and **TAB3** (containing HHTP linkers) show strong and sharp bands at 1241–1242 cm^−1^ of C−O stretching vibrations which are highly diagnostic of boronate ester formation [20]. In the case of **TAB2** and **TAB4** (containing THDMA linkers) the bands which can be assigned to C−O stretching of boronate groups were observed at 1217 and 1208 cm^−1^, i.e., slightly below the referential range (1240–1220 cm^−1^). However, this is in agreement with the values found for other systems based on anthracene tetraols [14]. An additional evidence for the boronate ester formation is the presence of medium strong bands in the range of 655 to 665 cm^−1^. It has been noted previously that the vibrational mode between 633 and 658 cm^−1^ (involving out-of-plane displacements of boron atoms that are *syn* to out-of-plane displacements of aryl hydrogen atoms) is diagnostic for boronate esters [21]. Importantly, the presence of THF is indicated by strong bands of C–O stretchings (except for **TAB3** where the intensity is medium) in the range of 1059 to 1070 cm^−1^. Specifically, the spectrum of **TAB3** shows the medium strong and broad band with maximum at 3288 cm^−1^ which indicates the presence of a significant amount of H-bonded OH end groups from HHTP linker. We suppose that H-bonding interactions may be responsible for a different morphology of **TAB3** observed by SEM and manifested by stronger agglomeration with respect to remaining materials. Finally, the presence of B(OMe)_2_ end groups should also be taken into consideration but it seems that they are generally absent based on the analysis of recorded FT-IR spectra.

### 3.3. Sorption Properties

**Figure 3:**
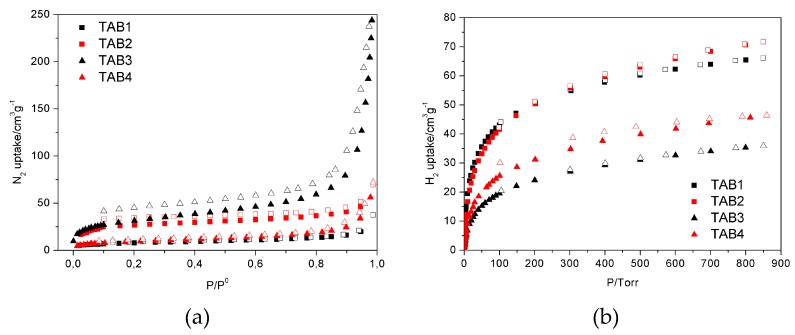
N_2_ (**a**) and H_2_ (**b**) sorption isotherms @77 K for materials **TAB1–4.**

**Figure 4:**
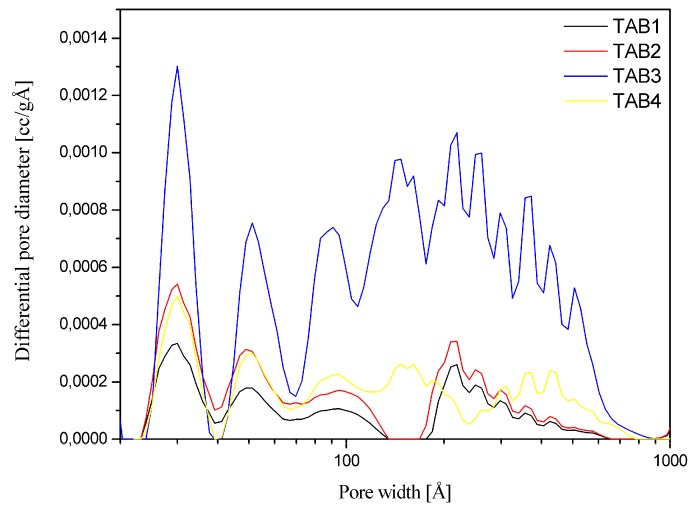
Pore size distribution (NLDFT model) for **TAB1–4** based on N_2_@77K sorption data.

The porosity of ionic materials **TAB1–4** was characterized using N_2_ gas adsorption at 77 K. Prior to the measurements, samples were heated at 150 °C under high vacuum (10^−3^ Torr) for 24 h. All studied materials exhibit similar type-II sorption isotherms with relatively slow and almost constant increase of sorption in the pressure range of 0.1−0.9 *P*/*P*_0_ (Figure 3a). In case of **TAB3** the N_2_ uptake reached ca. 250 cm^3^/g STP (*P*/*P*_0_ = 0.98), while it is rather weak in case of three remaining materials as it did not exceed 70 cm^3^/g STP (*P*/*P*_0_ = 0.98). We suppose that the higher N_2_ uptake for **TAB3** can be ascribed to its morphology which, according to SEM studies, is quite different from that observed for remaining three materials. It seems that **TAB3** possesses stronger meso- and macroporous character which results in a very distinct increase of nitrogen uptake from ca. 50 to 250 cm^3^/g in the *P*/*P*_0_ range of 0.8 to 1.0. The specificity of **TAB3** is well reflected by the pore size distribution (PSD) plot obtained using nonlocal density functional theory (NLDFT) model for N_2_ sorption (Figure 4). The relevant parameters including apparent surface area S_BET_ and the pore volume *V*_p_ at *P*/*P*_0_ = 0.98 were calculated (Table 1) based on Rouquerol’s consistency criteria [15], using the Brunauer–Emmett–Teller model.

In addition, the sorption of H_2_, CO_2_, and CH_4_ was studied. All materials exhibit type-I sorption of H_2_@77 K, which is not saturated at *P* = 850 Torr (Figure 3b). Remarkably, the materials **TAB1,2**, comprising tetraphenylborate nodes, have much higher H_2_ uptake exceeding ca. 70 cm^3^/g STP whereas for **TAB3,4** with larger tetrakis(4′-biphenylyl)borate units the H_2_ uptake reached 45 cm^3^/g STP, (*P* = 850 Torr). Comparison of the H_2_ uptake values for materials **TAB1,3** vs. **TAB2,4** indicates that the presence of THDMA linker is beneficial in this regard. Furthermore, the highest H_2_/N_2_ sorption selectivity was achieved for **TAB1** (ca. 5.2 for *P* = 500 Torr), which clearly indicates the prevailing microporous character of this material.

**Figure 5:**
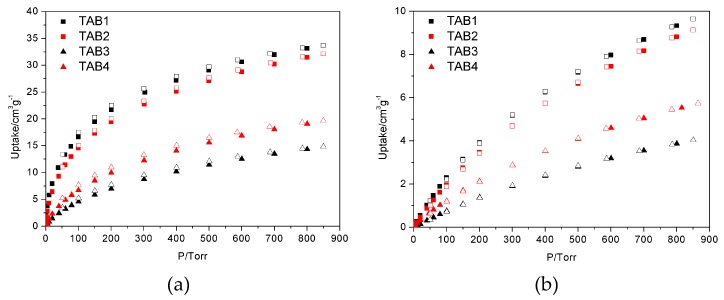
CO_2_ (**a**) and CH_4_ (**b**) sorption isotherms @273 K for materials **TAB1–4.**

It was hypothesized that the ionic structure of **TAB1–4** could be beneficial for the sorption molecules possessing polar bonds such as CO_2_. In fact, moderate CO_2_ sorption (up to ca. 35 cm^3^/g STP @273K, Table 1) was observed for **TAB1,2** (Figure 5a). The sorption of methane was much weaker and in all cases similar CO_2_/CH_4_ selectivities ranging from 3.4 to 3.7 were obtained (Figure 5b).

## 4. Conclusions

In conclusion, a family of ionic polymer networks, **TAB1–4**, based on tetraarylborate nodes with boronate linkages were prepared and characterized. PXRD analyses revealed that they possess amorphous structures. The morphology was studied by SEM revealing that obtained materials tend to form agglomerates composed of smaller nanoparticles whose appearance differs significantly in each case; specifically, the most regular spheroidal shape and uniform size of nanoparticles was observed for **TAB1**. ^6^Li MAS NMR analyses showed the presence of four-coordinate lithium cations located in the voids of anionic networks of **TAB1–4**. The study was aimed at evaluation of gas sorption selectivity of obtained networks. It was found that dinitrogen sorption is generally not high which leads to *S*_BET_ values not exceeding 115 m^2^/g. This may be due to the presence of solvated lithium cations which decreases the possibility for penetration of polymer networks with N_2_ molecules. Nevertheless, the sorption of dihydrogen is relatively good and in case of **TAB1** there is strong preference for sorption of H_2_ over N_2_, which shows the potential of such a material in gas separation. It seems that obtained materials possess a significant fraction of narrow micropores which can penetrated with dihydrogen molecules due to their small kinetic diameter (289 pm), but are not accessible for dinitrogen molecules (kinetic diameter of 364 pm) [22]. Overall, the obtained polymers have in fact a mixed character as the N_2_ uptake at low pressures is not strong but it increases very significantly at *P*/*P*_0_ close to 1 which indicates distinct meso- and even macroporosity.

## Figures and Tables

**Table 1:** Summary of surface areas *S*_BET_, gas uptakes (N_2_, H_2_), and total pore volumes *V*_p_ for **TAB1–4**.

Sorption parameter	TAB1	TAB2	TAB3	TAB4
*S*_BET_/m^2^ g^−1^	29.0	114.9	114.7	33.7
*V*_p_/cm^3^ g^−1^	0.058	0.107	0.402	0.111
N_2_@77 K/cm^3^ g^−1^ STP ^1^	37.4	69.4	259.7	72.0
H_2_@77 K/cm^3^ g^−1^ STP ^2^	66.1	71.7	35.9	46.4
CO_2_@273 K	33.7	32.1	14.8	19.7
CH_4_@273 K	9.6	9.1	4.0	5.7

^1^*P*/*P*_0_ = 0.98, ^2^*P* = 850 Torr.

## Supplementary Materials

The following are available online at https://www.mdpi.com/2073-4360/11/6/1070/s1. Figure S1–S4: NMR spectra of precursors **1,2**, Figure S5–S8: TGA plots of **TAB1–4**, Figure S9–S12: PXRD analyses of **TAB1–4**, Figure S13–S20: ^6^Li and ^11^B MAS NMR spectra of **TAB1–4**, Figure S21–S24: FTIR (ATR) spectra of **TAB1–4**, Figure S25, S26: PSD plots for **TAB1–4** (for N_2_ and H_2_ sorption).
