# Supplementary material for: Ionic Porous Organic Polymers Based on Functionalized Tetraarylborates
Source: Polymers (Basel). 2019 Jun 21;11(6):1070. doi: 10.3390/polym11061070 (PMC6630441; doi:10.3390/polym11061070)
Supplement: Supplementary file 1 [file polymers-11-01070-s001.pdf]

Supporting Information for Ionic Porous Organic Polymers Based on Functionalized Tetraarylborates

Patryk Tomaszewski, Marcin Wiszniewski, Krzysztof Gontarczyk,
Piotr Wieciński, Krzysztof Durka and Sergiusz Luliński[1,*]

[1]*Warsaw University of Technology, Faculty of Chemistry, Noakowskiego 3, 00-664 Warsaw, Poland*

1. NMR spectra of precursors 1-2.

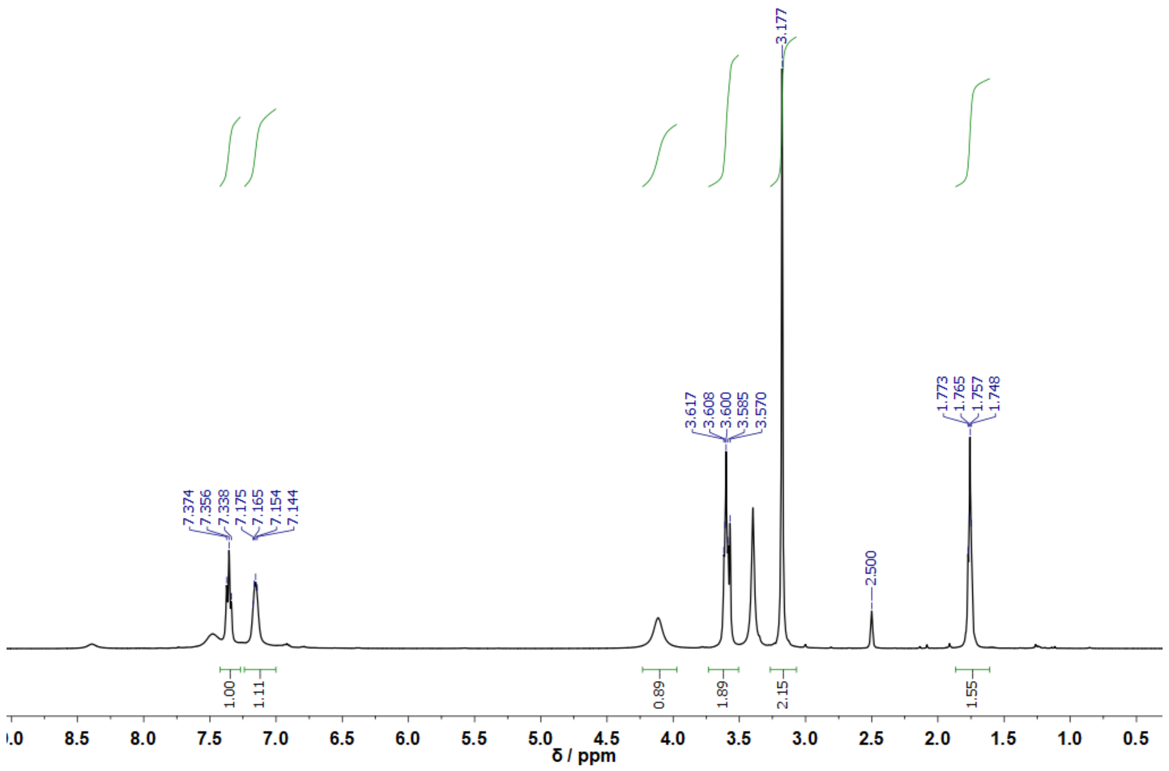

Figure S1. ^{1}H NMR (400 MHz, DMSO-d_6) spectrum of **1.**

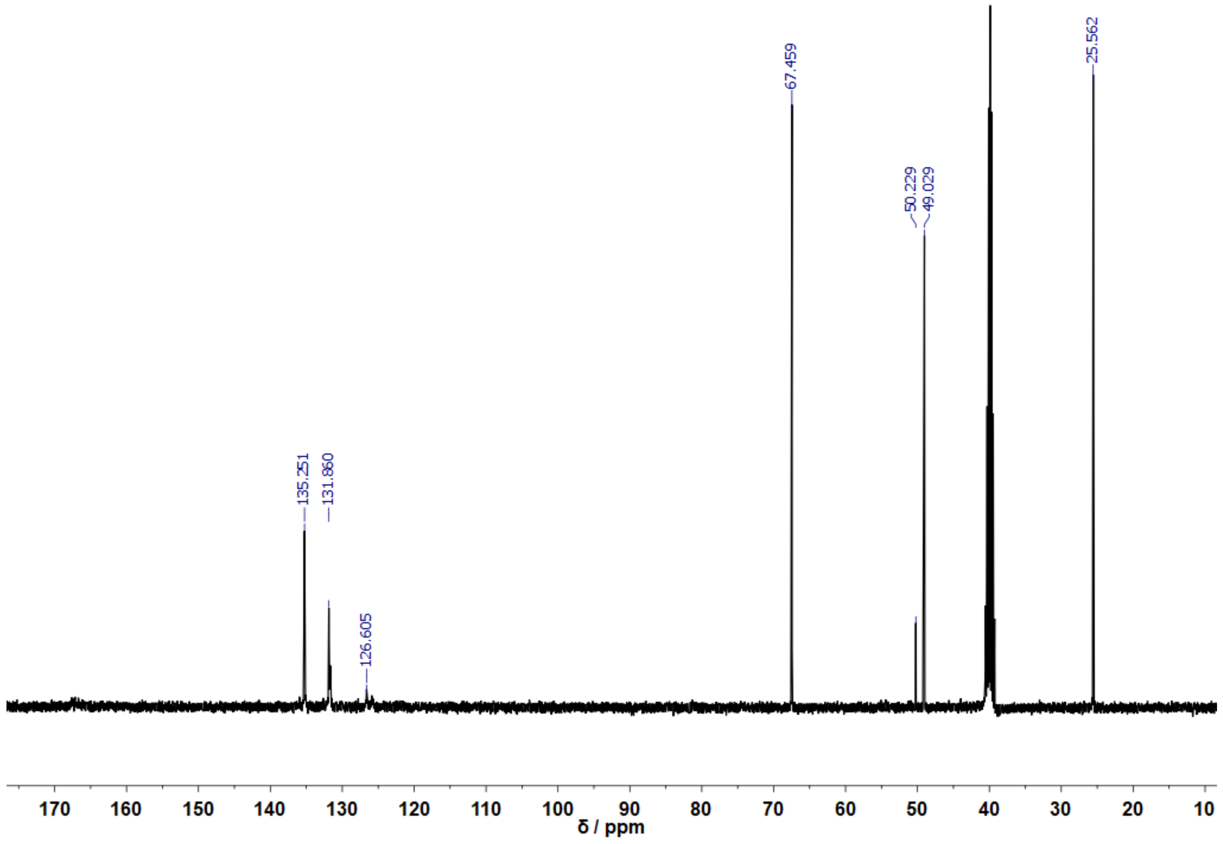

Figure S2. ^{13}C NMR (101 MHz, DMSO-d_6) spectrum of **1.**

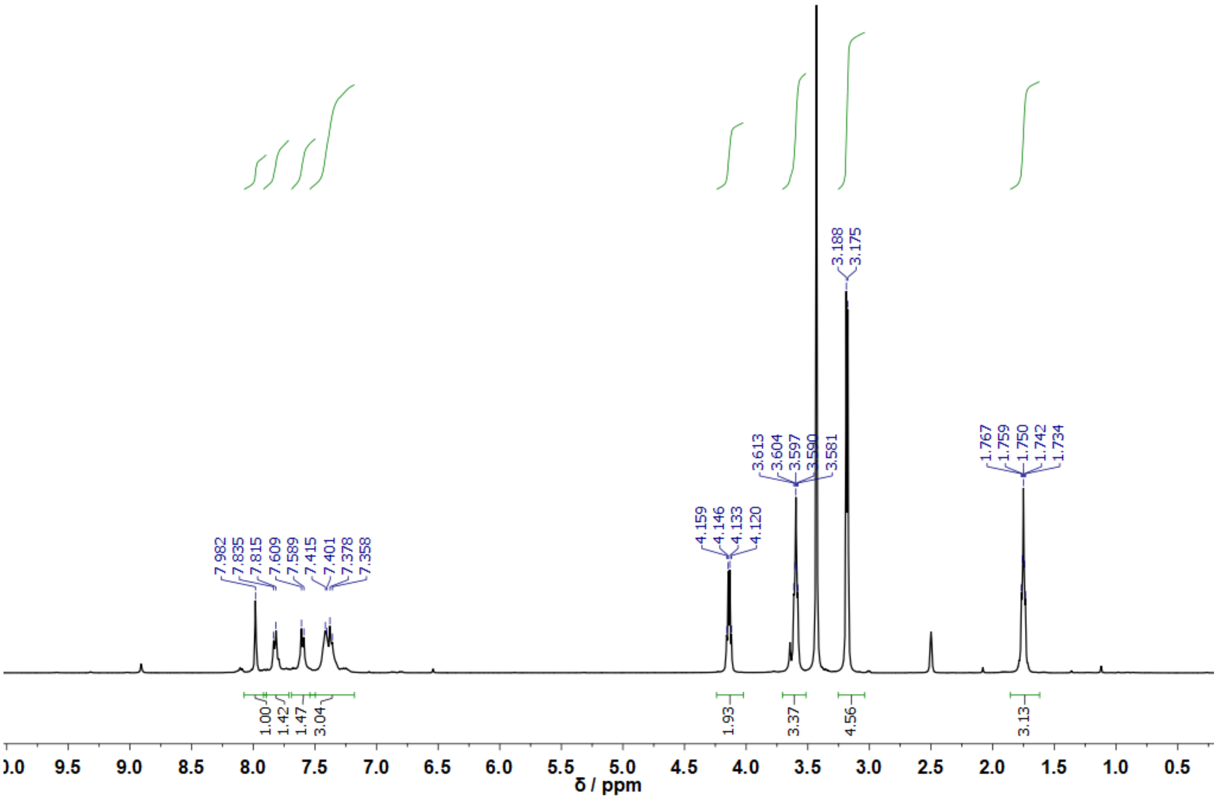

Figure S3. ^{1}H NMR (400 MHz, DMSO-d_6) spectrum of **2.**

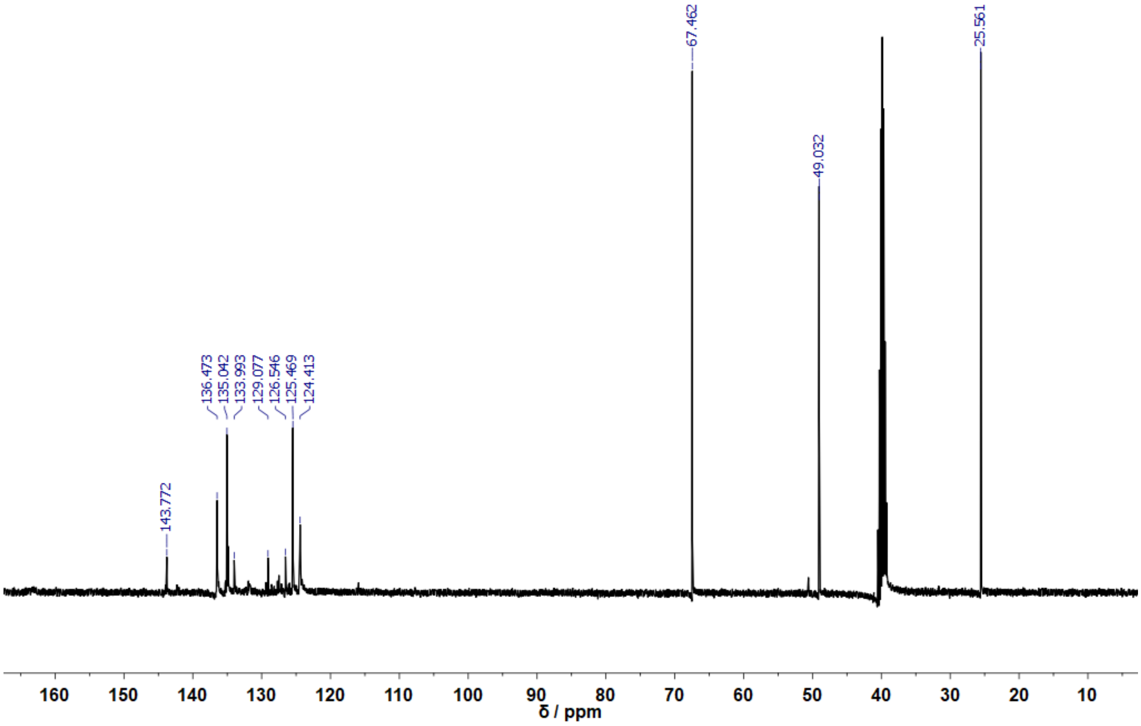

Figure S4. ^{13}C NMR (101 MHz, DMSO-d_6) spectrum of **2.**

2. MAS NMR spectra

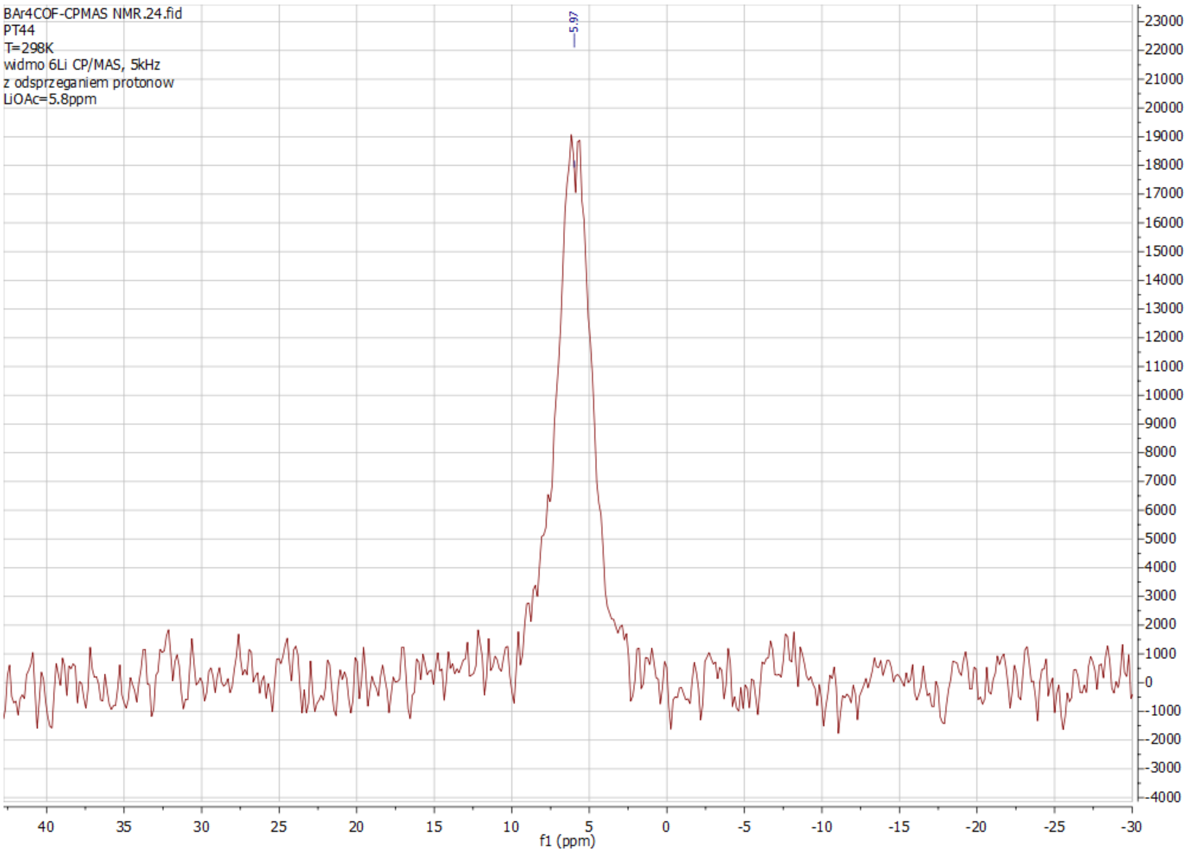

Figure S5. ^{6}Li MAS NMR spectrum of **TAB1**.

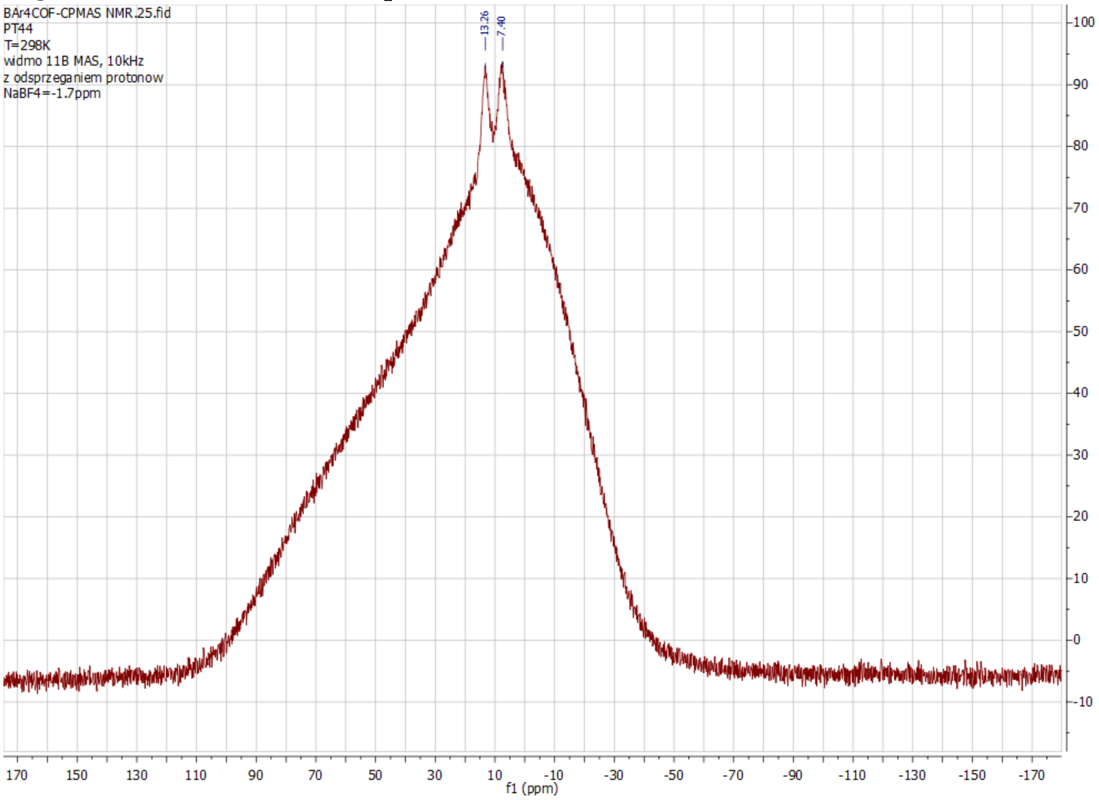

Figure S6. ^{11}B MAS NMR spectrum of **TAB1**.

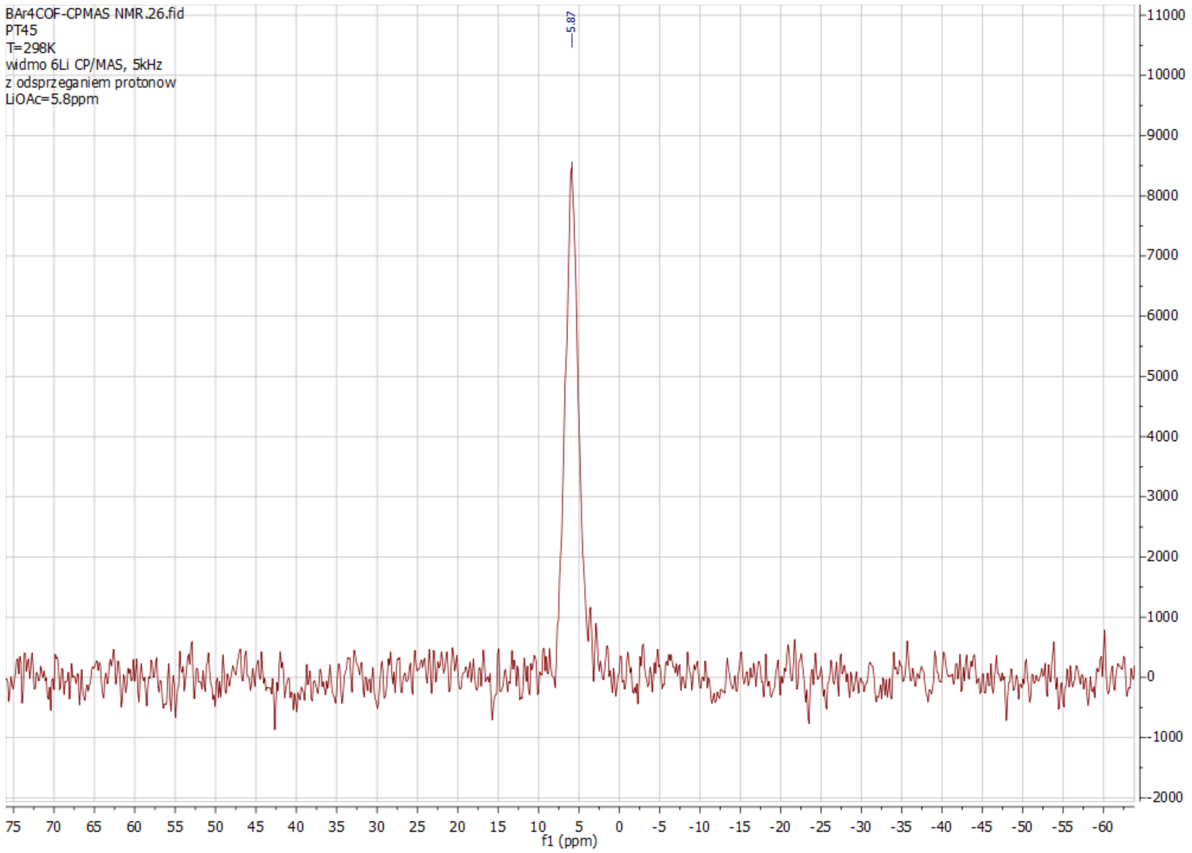

Figure S7. ^{6}Li MAS NMR spectrum of **TAB2**.

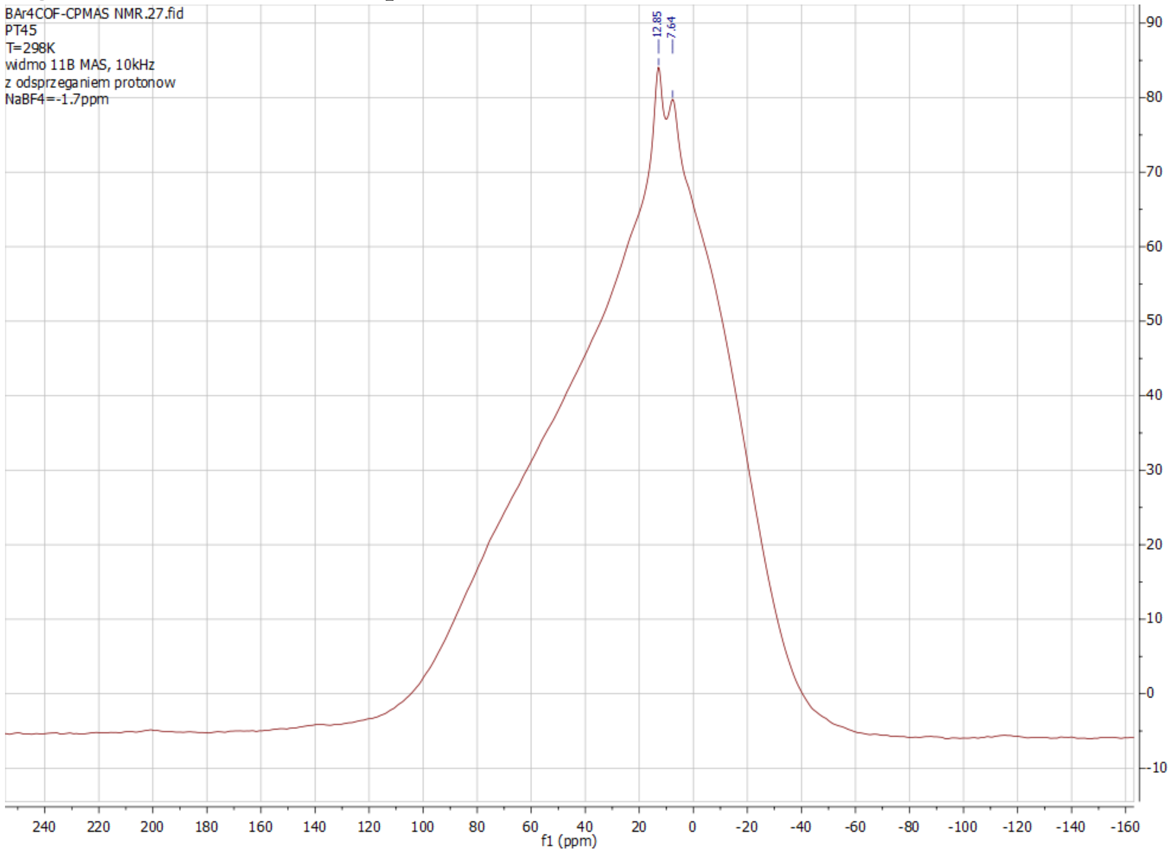

Figure S8. ^{11}B MAS NMR spectrum of **TAB2**.

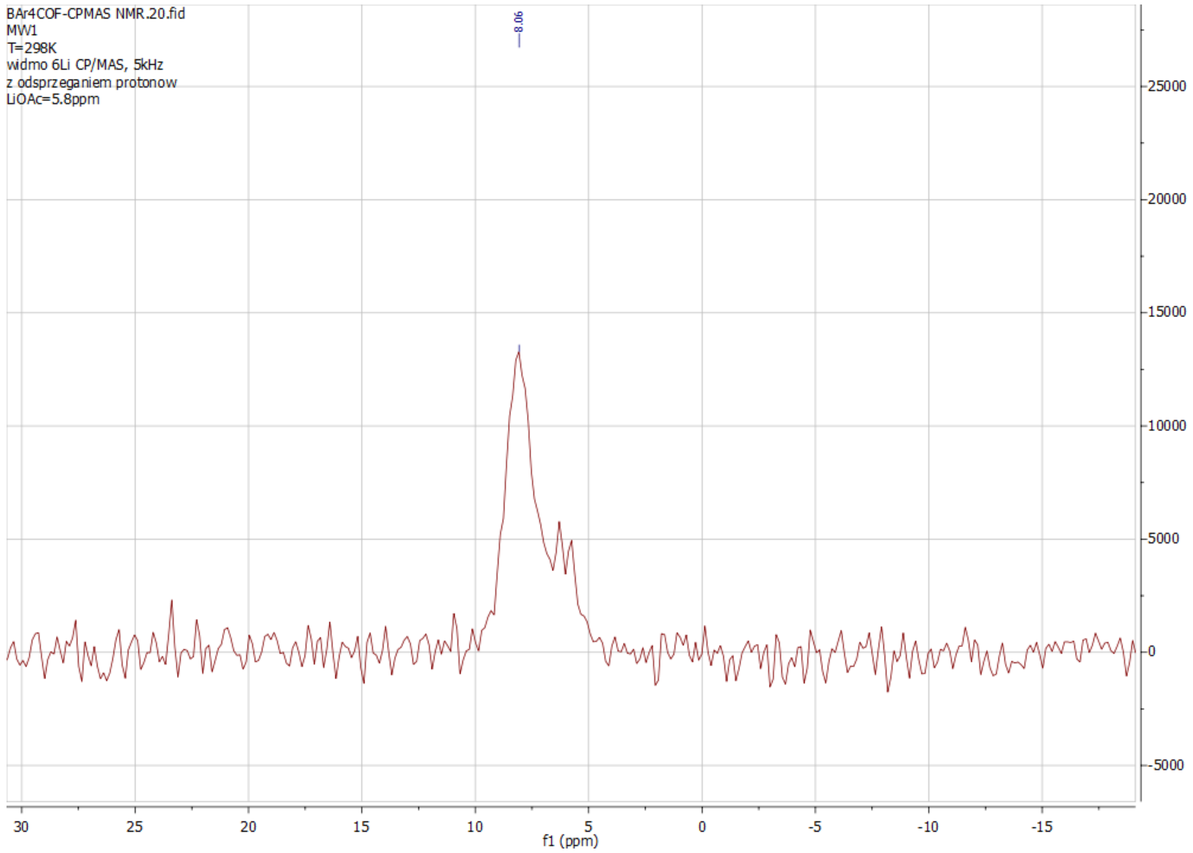

Figure S9. ^{6}Li MAS NMR spectrum of **TAB3**.

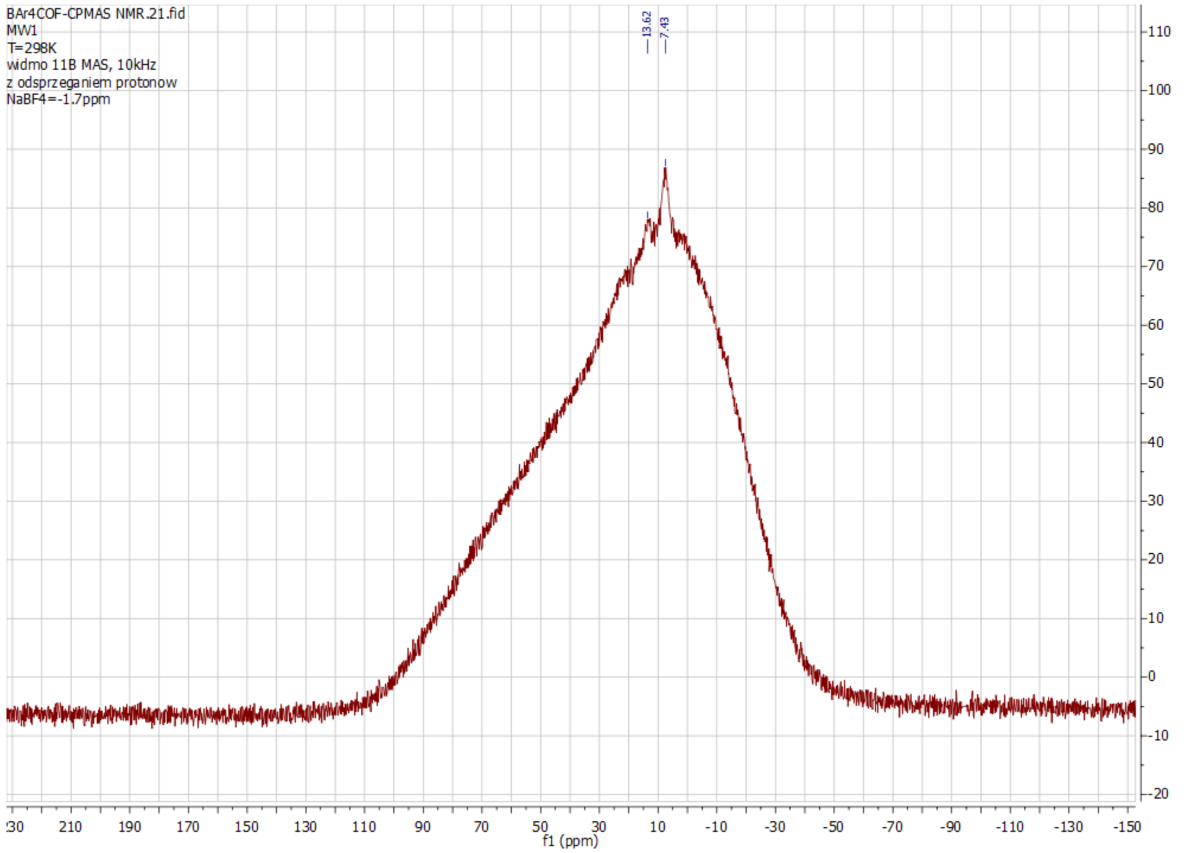

Figure S10. ^{11}B MAS NMR spectrum of **TAB3**.

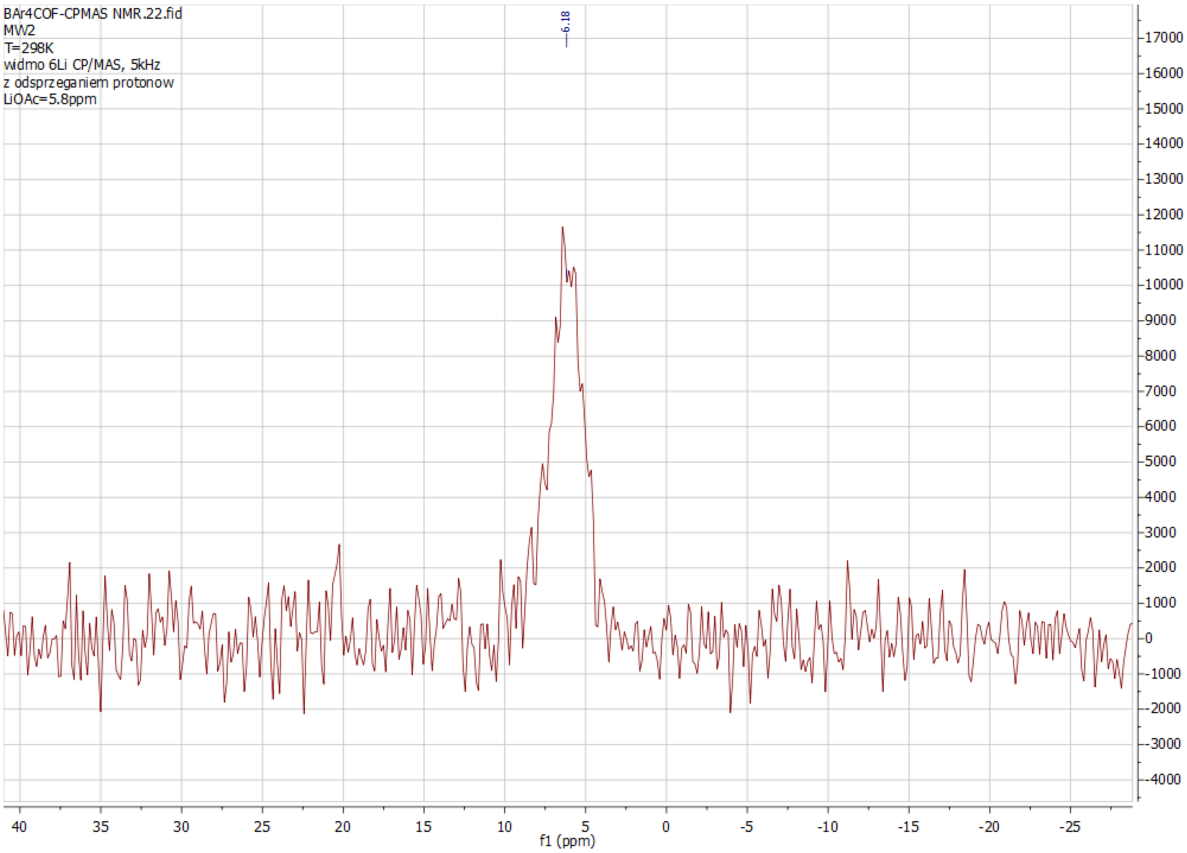

Figure S11. ^{6}Li MAS NMR spectrum of **TAB4**.

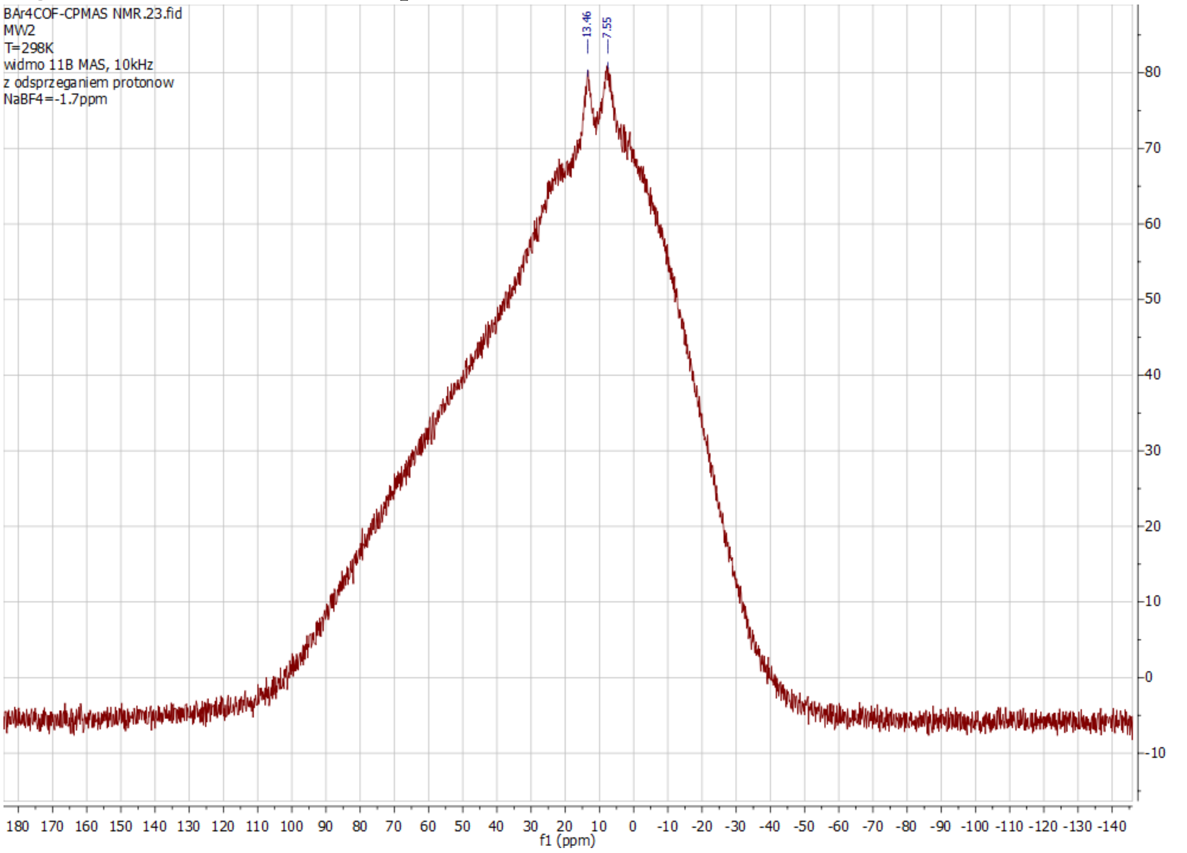

Figure S12. ^{11}B MAS NMR spectrum of **TAB4**.

3. FTIR (ATR) spectra

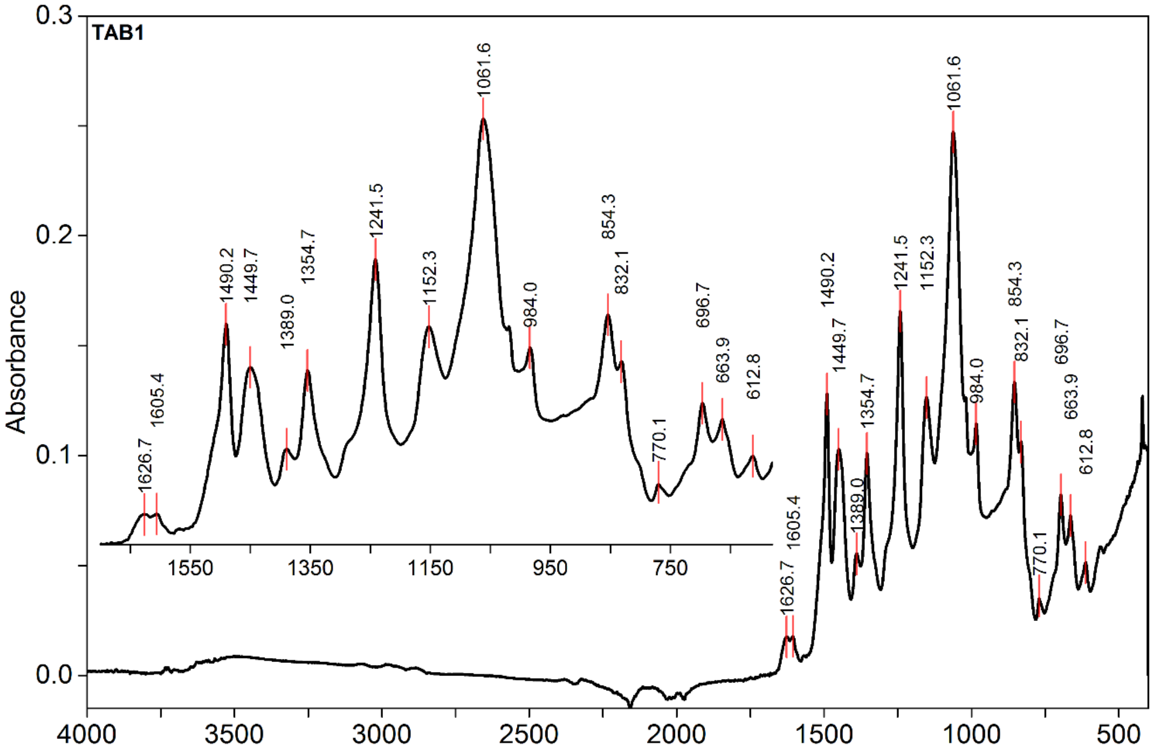

Figure S13. FTIR (ATR) spectrum of **TAB1**.

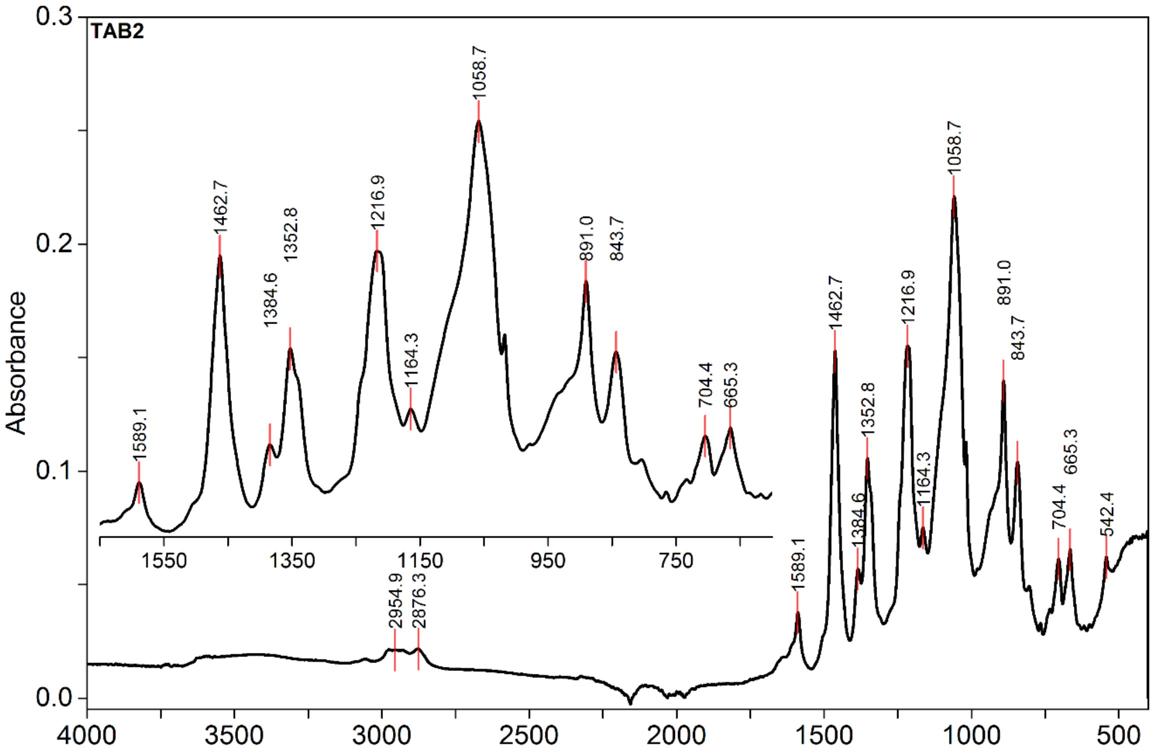

Figure S14. FTIR (ATR) spectrum of **TAB2**.

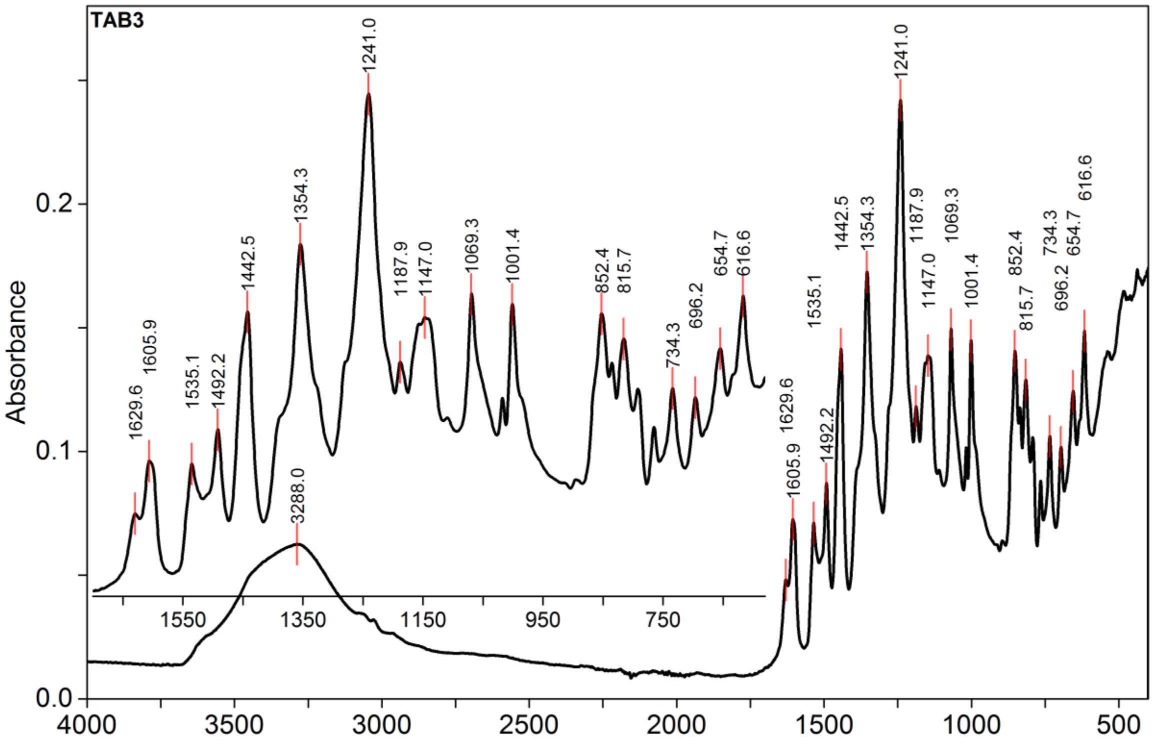

Figure S15. FTIR (ATR) spectrum of **TAB3**.

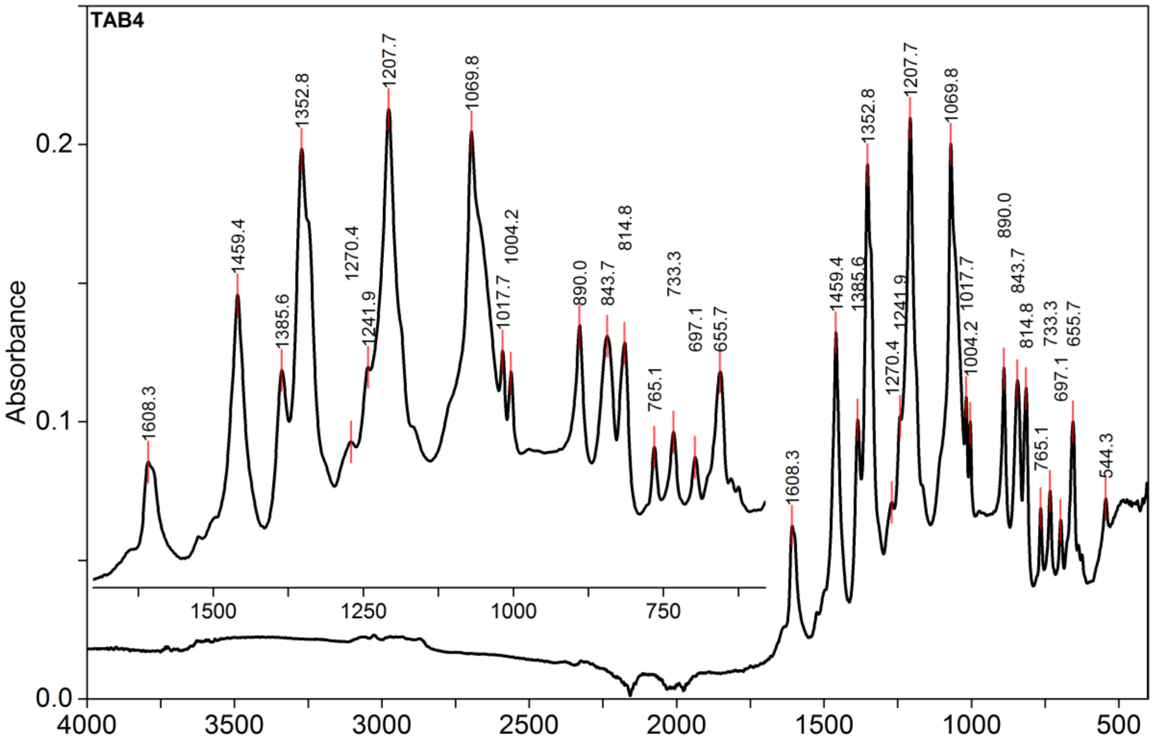

Figure S16. FTIR (ATR) spectrum of **TAB4**.

4. TGA analysis.

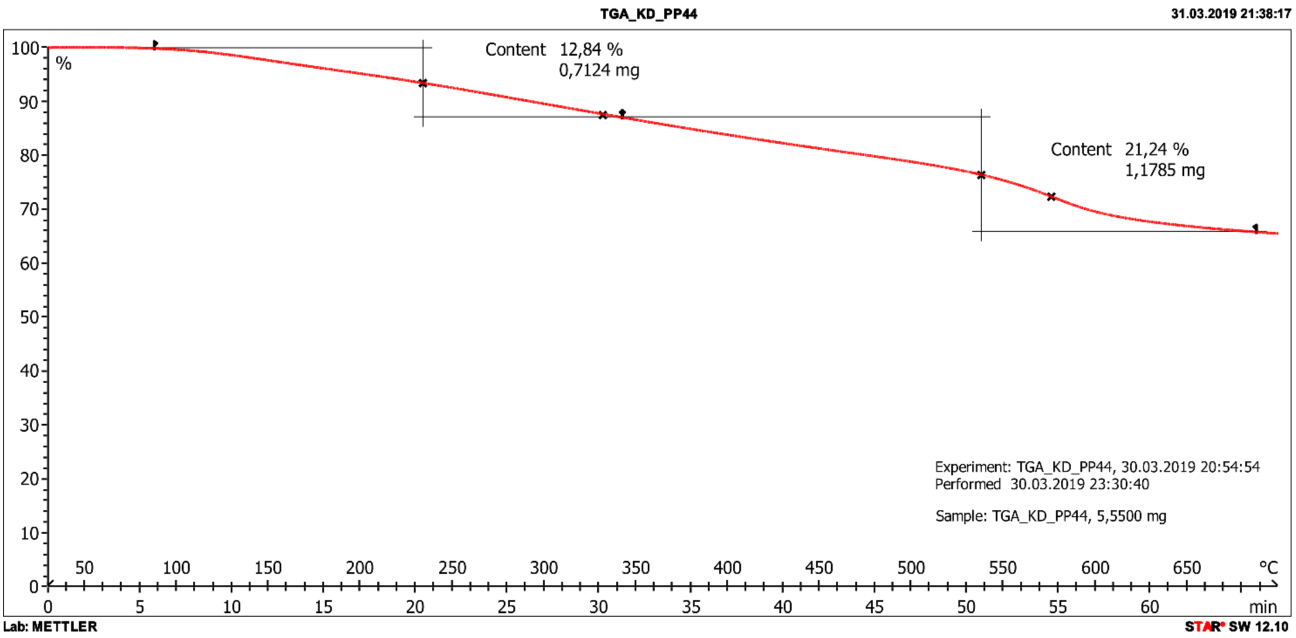

Figure S17. TGA plot of **TAB1.**

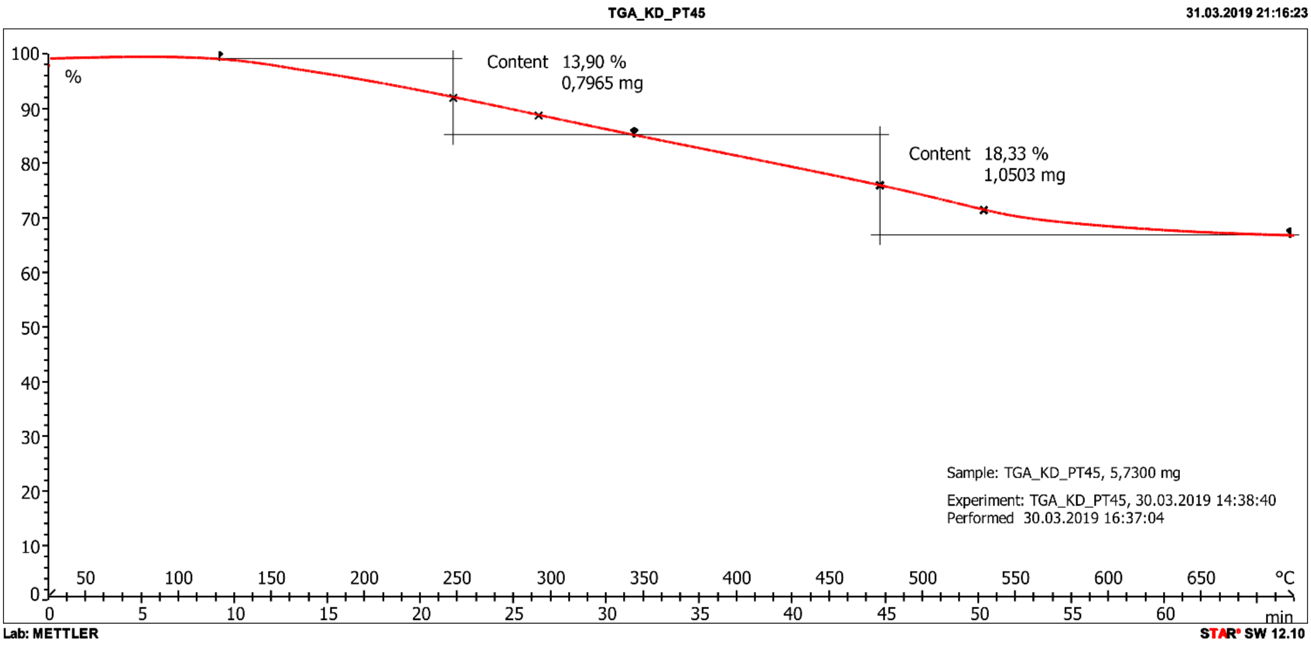

Figure S18. TGA plot of **TAB2**.

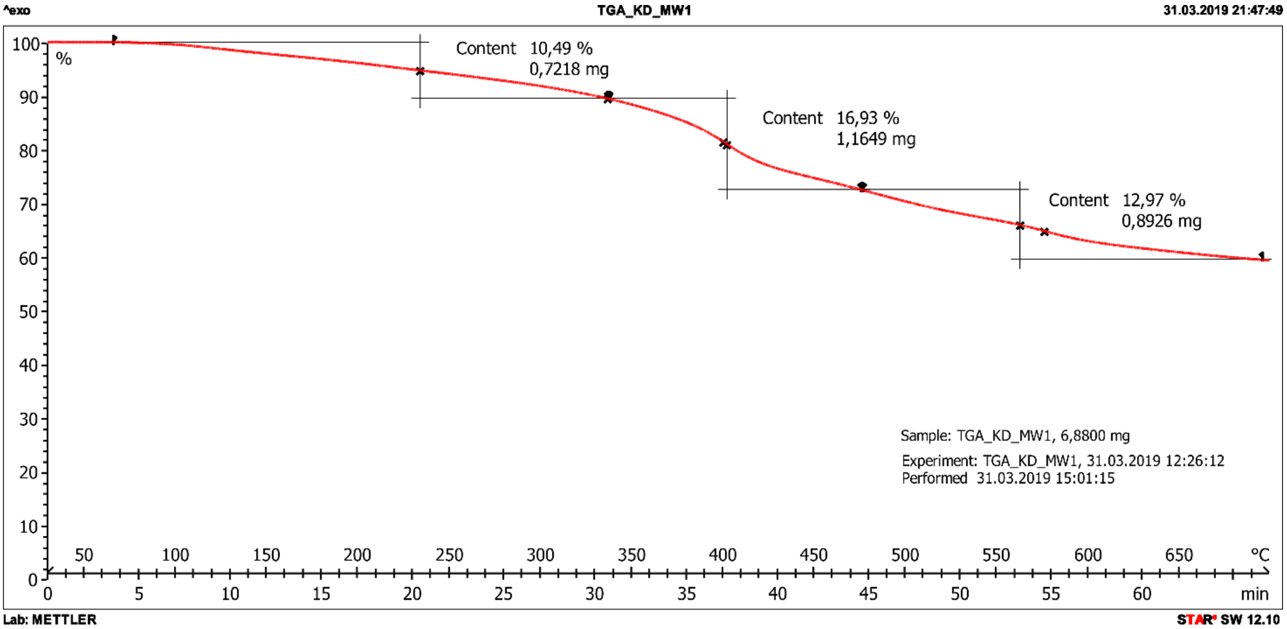

Figure S19. TGA plot of **TAB3**.

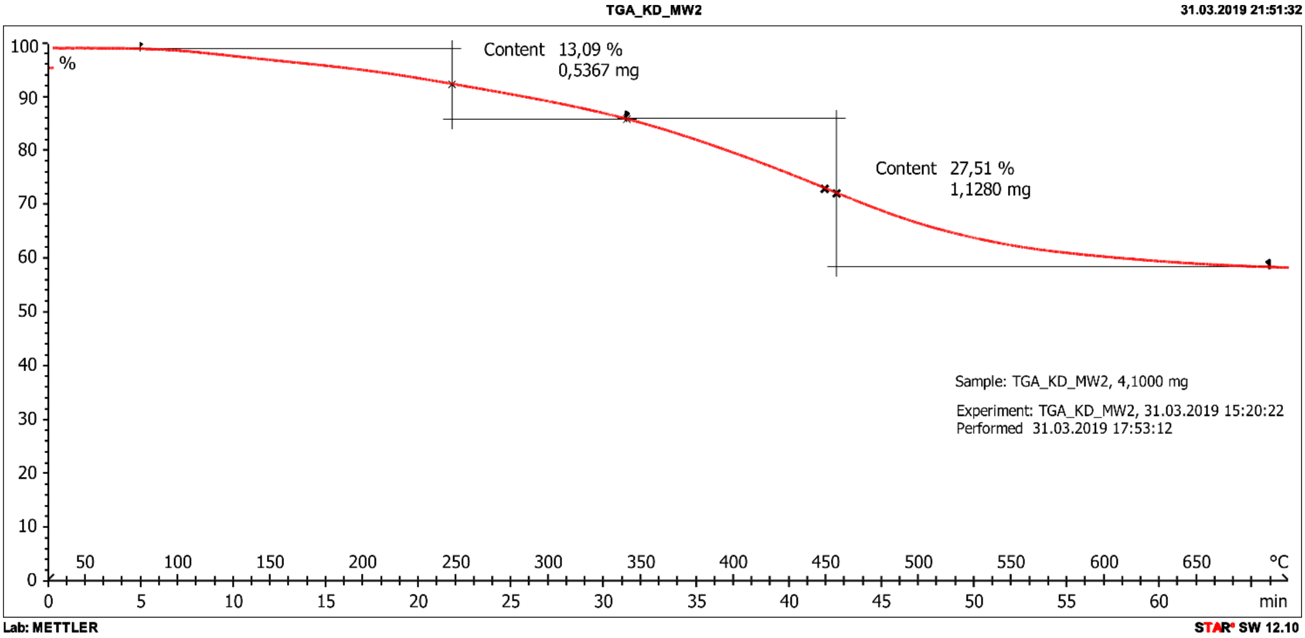

Figure S20. TGA plot of **TAB4**.
